# Inhibition of receptor for advanced glycation end-products (RAGE) improves alveolar fluid clearance and lung injury in a mouse model of acute respiratory distress syndrome (ARDS)

**DOI:** 10.1186/2197-425X-3-S1-A804

**Published:** 2015-10-01

**Authors:** R Blondonnet, J Audard, G Clairefond, C Belville, D Bouvier, L Blanchon, V Sapin, JM Constantin, M Jabaudon

**Affiliations:** CHU Clermont-Ferrand, Department of Anaesthesiology and Intensive Care Medicine, Clermont-Ferrand, France; Auvergne University, R2D2 - EA7281, Clermont-Ferrand, France; CHU Clermont-Ferrand, Department of Medical Biochemistry and Molecular Biology, Clermont-Ferrand, France

## Rationale

The receptor for advanced glycation end-products (RAGE) is a transmembrane multipattern receptor abundantly expressed on the basal surface of alveolar type (AT) I cells. RAGE is implicated in ARDS-associated alveolar inflammation [1,2], but its precise roles in lung injury remain unknown. It has been shown recently that RAGE axis could impact alveolar fluid clearance (AFC) through the modulation of epithelial sodium channels [3]. In mouse models of sepsis and of ARDS, treatment with anti-RAGE monoclonal antibody decreased mortality, and treatment with recombinant soluble RAGE (sRAGE, acting as a decoy receptor) was associated with improved lung injury.

## Objective

Using a murine model of ARDS, we evaluated whether RAGE modulation could regulate lung injury and AFC.

## Methods

60 anesthetised male C57BL/6JTj mice were divided in 4 groups; 3 of them underwent orotracheal installation of hydrochloric acid (day 0). Among these acid-injured mice, some were intravenously treated with an anti-RAGE monoclonal antibody (mAb) or intraperitoneal recombinant soluble RAGE (sRAGE). Mice from the Sham group underwent orotracheal instillation of saline and served as controls. At specified time-points (day 0, 1, 2 and 4), lung injury was assessed after a 30-minute period of mechanical ventilation by analysis of blood gases, alvolar permeability index, bronchoalveolar lavage (BAL) fluid content in interleukin (IL)-6 and AFC. AFC was calculated detecting changes into alveolar protein levels over time.

## Results

Acid-injured mice had higher permeability indexes, higher BAL IL-6 and marked hypoxemia on day 1 and 2, as compared with sham animals. AFC rates and PaO2/FiO2 ratios were higher in controls (35%/30min and 281 [262-319], respectively) than in HCl-injured mice on day 1 (8% and 181 [176-198], respectively, P < 0.0001) and day 2 (9% and 186 [174-205], respectively, P < 0.0001).

RAGE inhibition restored AFC on day 1 in both mAb-treated (8% versus 36%, p = 0.009) and sRAGE-treated (8% versus 37% p = 0,009) mice. RAGE inhibition significantly improved both PaO2/FiO2 ratio and permeability index on day 1, day 2, and anti-RAGE therapy could prevent increased BAL IL-6 levels on day 1 an day 2 in HCl-treated mice.

## Discussion

Our results support the efficacy of a RAGE inhibition strategy in improving AFC and lung injury in a translational mouse model of ARDS, and RAGE pathway may represent a therapeutic target during ARDS. Such findings should stimulate further research on the mechanistic links between RAGE pathway, AFC and lung alveolar injury and its resolution.

**Figure 1:**
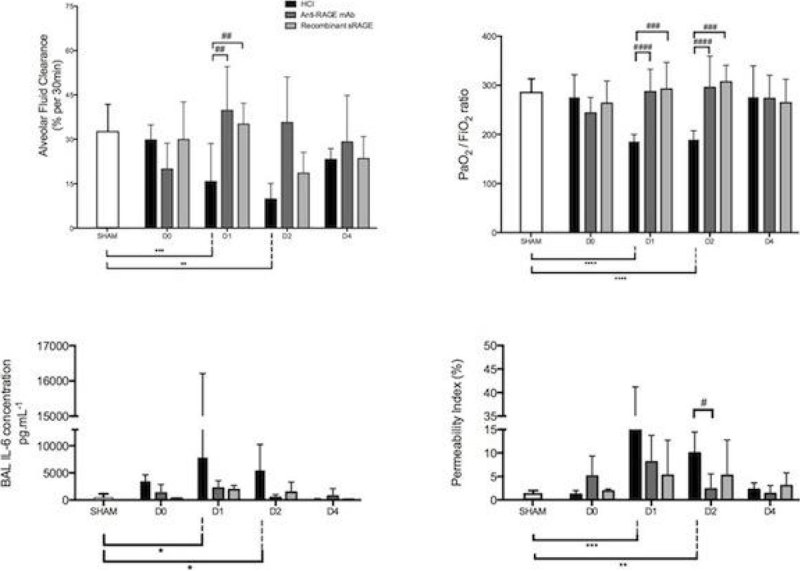
**Mice lung injury over time**.
